# Interoceptive attention and mood in daily life: an experience-sampling study

**DOI:** 10.1098/rstb.2023.0256

**Published:** 2024-08-26

**Authors:** Giulia L. Poerio, Megan Klabunde, Geoffrey Bird, Jennifer Murphy

**Affiliations:** ^1^ School of Psychology, University of Sussex, Falmer BN1 9QH, UK; ^2^ Department of Psychology, University of Essex, Wivenhoe Park, Colchester CO4 3SQ, UK; ^3^ Department of Experimental Psychology, University of Oxford, Oxford OX2 6GG, UK; ^4^ School of Psychology, University of Birmingham, Birmingham B15 2TT, UK; ^5^ Department of Psychology, University of Surrey, Guildford GU2 7XH, UK

**Keywords:** interoception, attention, emotion, experience-sampling methodology

## Abstract

Theories of emotion ascribe a fundamental role to the processing of bodily signals (interoception) in emotional experience. Despite evidence consistent with this, current knowledge is limited by a focus on interoceptive accuracy and laboratory-based interoception measures. This experience-sampling study examines how state interoceptive attention and state emotional experience are related in everyday life, providing the first data to our knowledge examining: (1) within-subject fluctuations in interoceptive attention across domains, and (2) the relationship between trait and state interoception. Compared with rates of exteroceptive attention (auditory attention: engaged 83% of the time), interoceptive signals captured attention approximately 20% of the time, with substantial within- and between-person variability across domains. There were relationships between interoceptive attention and emotion in daily life (greater attention being associated with more negative valence and fatigue) that were specific to interoceptive attention (different patterns were observed with exteroceptive attention). State measures of interoceptive (but not exteroceptive) attention were correlated with the trait interoceptive attention, but not accuracy. Results underscore the relationship between interoceptive attention and emotion, providing new insights into interoceptive attention and the structure of interoceptive ability. Future research should examine the source(s) of within- and between-person variability in interoceptive and exteroceptive attention and its relationship with emotional experience.

This article is part of the theme issue ‘Sensing and feeling: an integrative approach to sensory processing and emotional experience’.

## 1. Introduction

Almost every theory of emotion ascribes a fundamental role to the perception of bodily sensations (interoception) in emotional experience [1–6]. While it was proposed that emotions arise from the experience of bodily states alone [4], later evidence demonstrated that it is both experiencing a change in one's bodily state and the appraisal of the context that gives rise to the experience of emotion [5]. Although newer models with different theoretical perspectives have been introduced in recent years [3,6], all of these theories suggest that perceiving a change in one's bodily state is crucial for emotional experience. Importantly, growing evidence suggests that changes in bodily response are not a mere consequence of emotional experience but a foundational aspect of their instantiation (for a review see [7]).

This theoretical and empirical work has renewed interest in interoception [8], with both neuroimaging and behavioural evidence supporting links between the processing of internal bodily states and emotional experience. Although there is limited empirical evidence for a one-to-one correspondence between specific bodily states and distinct emotions, studies reliably show relationships between physiological activation and self-reported emotional experiences (e.g. disgust with cardiac and gastric activity; fear with increased heart rate and blood pressure; specific bodily parameters with emotional valence/arousal during ambulatory assessment) [9–13]. When asked to report on their experiences, people also tend to intuitively associate subjective emotional experiences with bodily sensations (e.g. disgust with digestive regions; anxiety with cardiac and respiratory regions) [14,15], and bodily sensations are a fundamental feature that define emotion concepts linguistically [16]. At the neural level, regions of the interoceptive cortex—such as the anterior insula and anterior cingulate cortex—have also been associated with the perception of one's own and others’ emotions, further suggesting a coupling between interoceptive and emotional experience (for reviews see [17,18]).

What follows from this evidence is that individual differences in the perception of internal bodily states (e.g. having better or worse interoceptive accuracy) should be related to individual differences in emotional experience and other emotional processes. Cardiac interoceptive accuracy (table 1), the focus of much research interest, is typically assessed by tasks that require participants to count their heartbeat over a series of internals (heartbeat counting) or by tasks that involve judgements of synchronicity between heartbeats and external stimuli (heartbeat detection; [19–24]). While the processes indexed by these tasks remain debated, both ostensibly assess whether participants' perceptions are a veridical representation of their actual heartbeats. Using these measures, laboratory-based studies consistently show that individual differences in cardiac interoceptive accuracy, assessed at a single time point, are related to differences in emotion regulation, emotional intelligence, emotional lability, emotion recognition and the intensity or arousal of experienced emotion (e.g. [18,25–28]; for a review see [17]). Other laboratory-based experimental measures of cardiac interoceptive accuracy assessed at one time point (heartbeat detection) have also been associated with naturally occurring emotional experiences examined in daily life (collected via experience-sampling; [29]; but see [30] for conflicting evidence using the heartbeat counting task). Newer evidence also suggests that cardiac interoceptive awareness—the extent to which an individual has insight into their cardiac interoceptive performance (table 1)—may also be related to emotional processing [31,32].

**Table 1.** Definition and measurement of interoceptive facets. ESM, experience-sampling methodology.

term	definition and measurement
interoceptive accuracy	Performance on behavioural tests that assess an individual's perception of their bodily state. This is most often assessed using cardiac interoceptive accuracy tasks (e.g. heartbeat detection or heartbeat counting tasks) that are influenced by both state and trait factors.
subjective interoceptive accuracy	An individual's beliefs regarding their ability to perceive their bodily states, measured via questionnaires or confidence ratings. Often questions ask about a range of interoceptive signals (e.g. accuracy of heartbeat, need to urinate etc.) and are then averaged. These are typically considered to pick up on trait factors, though may be influenced by state effects.
interoceptive attention	The amount of attentional resources an individual dedicates to their bodily states (e.g. the amount of time signals are the object of one's attention). Performance-based measures of interoceptive attention have yet to be developed.
subjective interoceptive attention	An individual's beliefs regarding their attention to internal signals, typically measured via questionnaires. Often questions ask about a range of interoceptive signals (e.g. attention to heartbeat, dry mouth, breathing). Questionnaires are thought to pick up on trait factors, though may be influenced by state effects. Where measured in the moment (e.g. via ESM), may be more influenced by state-factors.
interoceptive awareness	The amount of insight an individual has into their interoceptive ability, quantified by the correspondence between subjective and behavioural measures. This is sometimes referred to as ‘interoceptive insight’. To date, no research has examined the influence of state or trait factors on interoceptive awareness.

While the above evidence converges to suggest that the perception of bodily sensations strongly influences subjective emotional experience, much of the evidence base rests on the use of interoceptive tasks that have been argued to be invalid. Concerns regarding the validity of certain tasks assessing cardiac interoceptive accuracy (particularly the heartbeat counting task, which is affected by knowledge of heart rate; [33]) have shed doubts on evidence linking cardiac interoceptive accuracy to emotional processing. Indeed, inconsistent patterns of results do not neatly fit with theory and evidence linking better perception of bodily sensations to emotional experience. For example, there are gender differences in interoceptive accuracy and emotional processing, males displaying better interoceptive accuracy yet poorer emotional processing [34], and inconsistent findings have been reported regarding the relationship between self-reported emotional difficulties and aspects of interoception (e.g. [24,35–38]). It therefore remains unclear how important the processing of bodily states is for constructing emotional experience.

The limitations of previous methodological approaches may go some way toward explaining the aforementioned discrepancies. While laboratory and neuroimaging studies are vital for providing controlled, mechanistic evidence, current experimental paradigms are not optimized to comprehensively examine links between the processing of bodily states and emotional experience. For example, experiments typically focus on a single interoceptive signal (e.g. cardiac), despite evidence and theory suggesting that multiple bodily signals (e.g. respiratory, gastro-intestinal) are important for emotional experience (e.g. [10,14,39]). Experimental evidence also typically focuses on relating interoceptive accuracy scores collected offline (e.g. not during an emotion task) on one occasion to measures of emotion. While a valid approach, this provides little evidence that internal bodily *state* information is recruited in-the-moment for the experience, or construction, of emotional states as theoretically assumed. Trait-based measures, by definition, cannot capture potential fluctuations in either interoception or emotional experience and how these are related to each other [33,40]. More broadly, experimental approaches also lack ecological validity and do not always permit understanding of contextual influences or within-person variability on associations between interoception and emotional experience.

As well as a focus on laboratory- and trait-based measures, research linking individual differences in interoception to emotional experience has focused almost exclusively on interoceptive accuracy. However, to understand the role of interoception in emotional experience, it is important to consider not only the extent to which physiological changes are accurately perceived (i.e. level of accuracy) but, perhaps more importantly, also the extent to which they are focused on (i.e. *attended to*). Indeed, emerging evidence suggests that interoceptive accuracy and attention are separable constructs (e.g. [38,41]), with individual differences in interoceptive attention particularly relevant for understanding heightened bodily focus both within [42] and across different clinical populations [38]. More broadly, states of internal or external attention are well known to influence emotional experience, with attention a central component of models explaining the emotion generation process [43], and internal attention is considered necessary for bodily states to be interpreted as emotion [44,45]. For example, heightened self-focused attention is consistently linked to negative mood, depression and anxiety [46], and attention to internally generated thoughts (such as when the mind wanders) has both positive and negative emotional corollaries [47–49]. States of external attention characterized by absorption (e.g. immersive experiences in natural environments, and aesthetic experiences) are linked to positive emotions and heightened well-being [50–55] whereas biased attention towards threatening stimuli is thought to underlie anxiety disorders [56]. All of these studies underscore the importance of attention for the experience of emotion: where our attention is focused at any given moment—whether on the self, on thoughts, or on information coming from the external senses—is closely coupled to how we feel. Given both the role of attention and the often assumed importance of interoception in determining what and how we feel, it is perhaps surprising that research has not yet fully explored how interoceptive attention (rather than accuracy) is linked with emotional experience.

In the current study we sought to advance our understanding of interoceptive attention in daily life and its association with emotional experience, leveraging experience-sampling methodology (ESM). ESM is an ideal longitudinal approach for providing naturalistic evidence to complement and extend experimental work since it enables an examination of both interoceptive attention and emotional experience as states (rather than traits) that fluctuate over time within individuals. Thus, ESM can capture how changes in the environment (e.g. temperature changes, fluid intake, social context, activity levels) may differentially affect both individuals (e.g. some people may be more attentive than others to certain bodily states in some contexts—when exercising, or during an argument) and interoceptive signals (e.g. environmental temperature changes may affect attentiveness to hydration and temperature, but not pain or hunger), potentially resulting in different subjective emotional experiences. In the current study, participants were prompted 10 times daily for either 5 or 7 days to report on their interoceptive attention to 21 bodily signals, their state emotional experiences (fatigue, negative valence and tension), and, as a control, their attention to exteroceptive (specifically, auditory) sensations. Participants also completed trait measures of self-reported interoceptive accuracy [38] and attention [41], and measures of traits that predict the regular and frequent occurrence of certain emotions (e.g. anxiety and depression).

Our first aim was to describe the proportion of time people attend to interoceptive sensations and how this varies across interoceptive signals (e.g. gastric, cardiac, respiratory). While a previous study suggested substantial within-person variability in aspects of self-reported interoception [57], that study measured multiple aspects of interoceptive cognition (such as regulation of bodily states, the use of bodily states to infer emotional states) rather than interoceptive attention specifically. Where questions did probe attentional aspects of interoception (i.e. noticing, or attention regulation), they tended to conflate attention to sensations with subjective feelings (e.g. ‘I notice where in my body I feel uncomfortable’), making it difficult to disentangle attentional and emotional components of subjective reports. Furthermore, as questions focused on the body in general it remains unclear whether specific interoceptive signals are more or less attended to in daily life, or show different levels of variability within people. As such, our first aim was to examine interoceptive attention specifically and how this varied across interoceptive signals. We expected to observe substantial within-person variability in self-reported state interoceptive attention [57]. While we made no predictions regarding *which* states would capture more attention, we expected to find variability in attention to different bodily sensations.

Our second aim was to test whether fluctuations in state interoceptive attention are related to fluctuations in emotional experience at the same point in time. To our knowledge, ours is the first study to examine the relationship between interoceptive attention across multiple interoceptive signals and emotional experiences, over time in everyday life. Previous ESM studies have examined variation in emotional states (e.g. [58]) and their associations to non-interoceptive attentional states (e.g. [48,59–61]) as well as how behavioural cardiac interoceptive accuracy (heartbeat counting) relate to self-reported interoception [57]. Laboratory-based measures of cardiac interoceptive accuracy collected on one occasion have also been related to ESM measures of emotional experience [29]. However, only one recent study has measured *both* state interoception (hunger signals) and emotional experience with ESM [62]^1^. Using a time-lagged approach (e.g. hunger intensity at one time point predicting emotional state at a later time point), that study found bi-directional associations between hunger intensity and some positive emotional experiences. However, given the focus on lagged associations, it did not address how hunger and emotional experiences were related at the same point in time. If, as theoretical models propose, interoceptive processes are vital for in-the-moment generation of emotional states, then we would predict significant concurrent associations. Additionally, by focusing on one interoceptive signal (hunger intensity), the study did not address the contribution of attention to other bodily signals in the experience of emotion. Thus, our study was designed to provide a more comprehensive answer to the question of how attention across multiple interoceptive signals is related to concurrent emotional experience, by examining concurrent associations between attention to 21 interoceptive signals and three components of emotional experience. While we predicted significant concurrent associations between state emotional experience and state interoceptive attention, given mixed findings in the literature, we made no specific predictions regarding which aspect(s) of emotional experience (fatigue, negative valence and tension) would relate to interoceptive attention; however, we expected that at least one would be associated and that this would be specific to interoceptive attention (with a different relationship observed for auditory attention).

Our third aim was conceptual, specifically to examine the correspondence between trait- (dispositional and stable over time) and state- (in-the-moment and changeable over time) based measures of interoceptive attention. This is important for testing theoretical proposals regarding the structure of interoceptive abilities [64], and for examining the validity of trait-based measures given the biases inherent in retrospective self-reports [65]. In line with recent models and evidence that interoceptive attention and accuracy dissociate, we expected to find a significant positive association between state and trait measures of self-reported interoceptive attention, but no association between state or trait measures of interoceptive attention and trait interoceptive accuracy [41]. A supplementary analysis explored associations between trait anxiety and depression and both trait and state measures of interoceptive attention and trait interoceptive accuracy.

## 2. Method

### (a) Participants

Participants were 107 volunteers (*M*_age_ = 26.81, s.d. = 9.25, range: 18–57). The majority identified as female (75%) or male (23%), with the remainder identifying as non-binary (2%). The sample was predominately white (81%) and from the UK (81%). Participants were recruited to the study through research participation mailing lists; there were no incentives for taking part. Ethical approval was obtained from the University of Essex Science and Health Ethics Sub-committee (ETH 2021-1130).

### (b) Procedure

Participants completed a baseline survey which included trait measures (described below) and demographic questions. This was completed prior to the ESM training session. Individual and group training sessions were conducted online (maximum of 5 participants), during which the researcher explained the experience-sampling protocol, demonstrated what the experience-sampling questions and response scales looked like, and answered any questions. At the end of the training session, participants were signed up to the SurveySignal platform [66] to receive SMS experience-sampling prompts which started the following day.

#### (i) Experience-sampling protocol

Participants completed state measures of interoceptive attention across 21 signals, auditory attention and mood (see electronic supplementary material) using experience-sampling [67] in which SMS messages were sent to participants' smartphones containing a link to an online Qualtrics questionnaire. Text messages were scheduled via SurveySignal software [66] to occur 10 times daily at quasi-random intervals between 09.00 and 21.00 for either 5 or 7 consecutive days.^2^ There was at least 30 min between consecutive text message prompts and the link expired after 30 min. In total 4041 state-level observations were captured from 107 participants, with an average of 43 observations per participant (range: 15–70), corresponding to an average response rate of 66% (s.d. = 19.11, range: 21–100%)

### (c) Trait measures

#### (i) Trait interoceptive attention

Trait interoceptive attention was assessed using the 21-item Interoceptive Attention Scale (IATS; [41]). The IATS is a trait-based measure of the extent to which sensations from various interoceptive signals capture attention (e.g. ‘Most of the time my attention is focused on whether I am hungry’). Responses are given on a 5-point Likert scale ranging from 1 (strongly disagree) to 5 (strongly agree). An overall average score was computed, where higher scores indicated higher levels of self-perceived interoceptive attention across all interoceptive signals. The internal consistency reliability of this scale, Cronbach's alpha, was good (*α* = 0.85)^3^.

#### (ii) Trait interoceptive accuracy

Trait interoceptive accuracy was assessed using the 21-item Interoceptive Accuracy Scale (IAS; [38]). The IAS is a trait-based measure of subjective beliefs regarding the accuracy of interoceptive perception for various interoceptive signals (e.g. ‘I can always accurately perceive when I am hungry’). Responses are given on a 5-point Likert scale ranging from 1 (strongly disagree) to 5 (strongly agree). An overall average score was computed, where higher scores indicated higher levels of self-perceived interoceptive accuracy across all interoceptive signals (*α* = 0.85).

#### (iii) Trait anxiety

Trait anxiety was assessed with 20 trait items of the state–trait anxiety inventory (STAI; [69]). Participants rated how they generally felt for each statement (e.g. ‘I feel nervous and restless') on a 4-point Likert scale from 1 (almost never) to 4 (almost always). Negatively worded items (e.g. ‘I am "calm cool and collected"’) were reverse scored and all items were summed to provide an overall score, where higher scores indicated greater trait anxiety (*α* = 0.94).

#### (iv) Trait depression

Depression was assessed with the 13-item Moods and Feelings Questionnaire [70]. Participants rated how they had been feeling or acting in the past two weeks (e.g. ‘I felt miserable or unhappy’) on a 3-point Likert scale from 1 (not true) to 3 (true). Items were summed to provide an overall score where higher scores indicated greater trait depression (*α* = 0.89).

### (d) State experience-sampling methodology, measures

#### (i) State interoceptive attention

The IATS was modified to capture the extent to which interoceptive attention across 21 interoceptive signals was the object of attention over the preceding 5 min. Participants were asked ‘In the last 5 min, about how much of the time were you paying attention to the following bodily signals … ’, with each signal represented as a single item (e.g. ‘whether my heart is beating fast’, ‘whether I need to urinate’). We adapted the 21-item IATS for two reasons; first, to ensure the matching of sensations across state and trait measures (the IAS and IATS assess the same interoceptive signals; [41]); and, second, asking about multiple interoceptive signals allowed us to examine differences in attention across bodily states. Each item was rated on a 5-point scale (1 = none of the last 5 min, 2 = some of the last 5 min, 3 = about half of the last 5 min, 4 = most of the last 5 min, 5 = all of the last 5 min). Participants were reminded that they should (1) rate interoceptive attention not accuracy, (2) rate signals regardless of whether they were ‘truly’ occurring and (3) rate attention to feelings coming from inside their body. Scores for each question were averaged to provide an overall score for state interoceptive attention, where higher scores indicated more attention paid to bodily signals over the preceding 5 min.

#### (ii) State auditory attention

Participants rated attention to auditory signals in their environment over the preceding 5 min using a single item ‘In the last 5 min, how much of the time were you paying attention to auditory signals?’, using the same scale as for state interoceptive attention. Participants were told that ‘auditory signals include things like listening to music, the television or a podcast as well as things in the background like birds singing, transport noises, the sound of people talking’ and that they should rate their attention regardless of whether the signal was present (e.g. thinking about whether you heard a noise, even if you realize there was no noise, would still count as paying attention to an auditory signal). This exteroceptive control question was included to test the possibility that associations between interoceptive attention and mood might result from general attentional processes rather than interoception specifically. We opted to use auditory attention as a control over other sensory modalities as there is debate regarding whether certain sensory modalities are fully separable from interoception (e.g. taste, touch, smell; [17]) and because during waking hours visual input is near-constant, which would have likely resulted in a restricted distribution of scores if visual attention was used as a control.

#### (iii) State emotional experience

Emotional states over the preceding 5 min were measured using a scale to capture dimensions of core affect validated for use in experience-sampling studies [71]: valence (i.e. the extent to which emotional experience is positive or negative) and arousal (i.e. the extent to which emotional experience is activating or deactivating). The authors of [71] distinguish between two aspects of arousal: (1) tense arousal (relaxation–tension) and (2) energetic arousal (tiredness–wakefulness). In the present study, participants rated six bipolar items on 7-point scales: tired–awake, content–discontent, agitated–calm, full of energy–lacking in energy, unwell–well, relaxed–tense. Three items (unwell–well, tired–awake and agitated–calm) were reverse-coded and scores were created for: (1) negative valence (average of content–discontent and reverse of unwell–well)—here higher scores indicate more negative valence; (2) tension (average of relaxed–tense and reverse of agitated–calm)—here higher scores indicate more tense arousal; and (3) fatigue (average of full of energy–lacking in energy and reverse of tired–awake)—here higher scores indicate lower levels of energetic arousal/higher fatigue. We opted to focus on core affect (valence/arousal) to capture emotional experiences rather than discrete emotions (e.g. sadness) since bodily information may not map onto specific feeling states or may map to multiple states (e.g. a racing heart rate may indicate anxiety, excitement or fear). We chose to use averaged scores for each of the three core affect dimensions, rather than analysing each item separately, given recommendations not to use single items to assess complex constructs in experience-sampling studies (e.g. [72]) and also because the two-item versions have been psychometrically validated for assessing within-person fluctuations in core affect over time (see [71] for details).

## 3. Results

Our first aim was to examine the proportion of time people attend to interoceptive sensations and how this varies across interoceptive signals (e.g. gastric, cardiac, respiratory) as well as within people over time. Table 2 shows the responses for attention to interoceptive and auditory signals across all observations. It shows the percentage of observations in which the signal was the object of attention (i.e. 'yes' versus 'no'), as well as the extent to which that signal was the object of attention (columns for ‘some’, ‘half’, ‘most’, ‘all’). Some form of interoceptive attention was engaged only 20% of the time whereas auditory attention was engaged 83% of the time (reflected by the proportion of ‘yes’ responses for interoceptive and auditory attention items, respectively; table 2). The most commonly engaged interoceptive signals were thirst (57% of the time), hunger and body temperature (44% of the time), muscles (34% of the time), tastes (31% of the time), urination (29% of the time), pain (24% of the time), heart beating (18% of the time) and breathing fast (17% of the time). These results suggest that in daily life attention to interoceptive information is substantially lower than attention paid to auditory signals in the environment. Indeed, comparing means showed that in daily life participants were significantly more attentive to auditory (*M* = 2.84, s.d. = 0.90) than to interoceptive information (*M* = 1.32, s.d. = 0.21), *t*_106_ = 18.91, *p* < 0.001, *d* = 1.83.

**Table 2.** Interoceptive and auditory attention rates in daily life (4041 level-1 observations from 107 participants).

	attention paid over the preceding 5 min (*n* = 1041)					
	**% yes**	**% no**	**% some**	**% half**	**% most**	**% all**
**interoceptive attention**	20	80	13	4	2	1
**auditory attention**	83	17	33	15	15	20
**interoceptive signal**						
thirst	57	43	35	13	6	3
hunger	44	56	23	9	7	5
temperature	44	56	26	10	5	3
muscles	34	66	21	8	3	2
tastes	31	69	19	6	4	3
urination	29	71	19	5	3	2
pain (not injury)	24	76	14	5	3	2
heart beating	18	82	12	3	1	1
breathing fast	17	83	12	4	1	0
itch	13	87	9	3	1	1
defaecation	13	87	8	2	1	1
pleasant touch	11	89	7	2	1	1
hurt (after injury)	11	89	7	2	1	0
sexual arousal	10	90	7	1	1	2
wind	10	90	8	1	0	0
cough	9	91	7	1	0	0
blood sugar	9	91	7	1	1	0
nauseated	8	92	6	1	0	0
sneeze	8	92	6	1	0	0
tickle	8	92	6	1	0	0
burp	6	94	5	1	0	0

### (a) Within- versus between-person variation in interoceptive attention, auditory attention and mood

To explore the extent to which state variables vary within versus between individuals we calculated the Intraclass Correlation Coefficient (ICC). This was calculated from the unconditional model (random intercept models with the dependent variable and no predictors) by dividing the random-effect variance by the total variance (sum of random effect and residual variance). This value can be interpreted as the proportion of variance in a dependent variable attributable to differences between people, with the remainder attributed to variation within people [73]. Table 3 shows the ICC for each variable measured with corresponding percentages attributable to within-person variability; 44% of the variance in interoceptive attention was attributable to within-person differences, compared with 62% with auditory attention. Of the interoceptive modalities, sexual arousal, pain, itch, muscles and temperature had the lowest levels of within-person variability and wind, blood sugar, sneeze and hunger showed the highest. Negative valence and tense arousal showed substantial within-person variability, and fatigue showed an even greater degree of within-person variability. One plausible interpretation is that variables with higher ICC values may be more stable and ‘trait-like’ (they vary between people but vary less within a person on different occasions) than those with lower ICC values, which may be more ‘state-like’ (they vary little on average among people but vary more across different occasions within a person), perhaps because they are more dependent upon other states or subject to contextual influence.

**Table 3.** Intraclass-correlation coefficients (ICC) and within-person variability percentages for state measures of interoceptive attention, auditory attention and emotional experience.

	**ICC**	**% within-person variability**
**interoceptive attention**	0.56	44
**auditory attention**	0.38	62
**interoceptive signal**		
sexual arousal	0.51	49
pain (not injury)	0.43	57
itch	0.40	60
muscles	0.39	61
temperature	0.38	62
thirst	0.33	67
hurt (after injury)	0.33	67
defaecation	0.31	69
heart beating	0.30	70
breathing fast	0.29	71
tickle	0.24	76
cough	0.24	76
burp	0.21	79
urination	0.21	79
nauseated	0.19	81
tastes	0.19	81
pleasant touch	0.17	83
wind	0.14	86
blood sugar	0.14	86
sneeze	0.14	86
hunger	0.13	87
**state mood**		
negative valence	0.51	49
tense arousal	0.41	59
fatigue	0.28	72

### (b) Multi-level models

Subsequent aims were addressed with multi-level modelling [74] using the Mixed procedure in IBM SPSS software. The data had a two-level structure in which responses collected over a series of time points (level-1 units) were nested within individuals (level-2 units). The within- and between-subjects variance of the dependent variable was partitioned by fitting random intercept terms for each individual. Slopes as well as intercepts were allowed to vary when doing so produced a better fit to the data. The non-independence of observations within individuals was modelled by fitting an autoregressive correlation structure to event-level residuals. Level-1 predictors were group-mean centred and level-2 predictors were grand-mean centred.

### (c) State interoceptive attention predicting concurrent emotional experience

Our second aim was to explore concurrent relationships between state interoceptive attention (averaged across the 21 interoceptive signals) and emotional experience, while accounting for state auditory attention. Both state attention variables (interoceptive and auditory), which were significantly positively correlated when averaged across time points (*r*_107_ = 0.44, *p* < 0.001), were included as simultaneous independent predictors of emotional experience with a separate model for each mood state as the dependent variable.^4^

State interoceptive attention was a significant positive predictor of negative valence (*b* = 0.29, s.e. = 0.10, *t*_67_ = 2.83, *p* = 0.006, 95% confidence interval (CI) [0.09, 0.49]) and marginally significant for fatigue (*b* = 0.29, s.e. = 0.15, *t*_78_ = 1.86, *p* = 0.067, 95% CI [−0.02, 0.59]) but not tense arousal (*b* = 0.18, s.e. = 0.13, *t*_62_ = 1.37, *p* = 0.177, 95% CI [−0.08, 0.43]). State auditory attention showed distinct relationships to emotional experience; it was a significant negative predictor of negative valence (*b* = −0.08, s.e. = 0.02, *t*_66_ = −5.02, *p* < 0.001, 95% CI [−0.11, −0.05]), tense arousal (*b* = −0.07, s.e. = 0.02, *t*_58_ = −4.48, *p* < 0.001, 95% CI [−0.11, −0.04] and fatigue (*b* = −0.10, s.e. = 0.03, *t*_78_ = −3.87, *p* < 0.001, 95% CI [−0.15, −0.05]). Interested readers wishing to see relationships between attention to specific interoceptive signals and concurrent mood can view these analyses in the electronic supplementary material.

### (d) Trait measures predicting state interoceptive attention

Our third aim was to explore how trait measures of interoceptive attention, interoceptive accuracy, and emotion (anxiety and depression) are related to state interoceptive attention. We used a multi-level regression model in which all the trait variables (interoceptive attention, interoceptive accuracy, depression and anxiety) were entered as simultaneous level-2 independent variables predicting level-1 state interoceptive attention.

State interoceptive attention was significantly positively predicted by trait interoceptive attention (*b* = 0.20, s.e. = 0.04, *t*_101_ = 5.61, *p* < 0.001, 95% CI [0.13, 0.27]) but not by trait interoceptive accuracy (*b* = 0.02, s.e. = 0.04, *t*_100_ = 0.55, *p* = 0.583, 95% CI [−0.06, 0.11]), trait anxiety (*b* = −0.0005, s.e. = 0.003, *t*_100_ = −0.21, *p* = 0.883, 95% CI [−0.006, 0.004]), or trait depression (*b* = 0.002, s.e. = 0.005, *t*_101_ = 0.36, *p* = 0.721, 95% CI [−0.008, 0.01]). This suggests a level of convergence between trait and state measures of the same construct (interoceptive attention) and, at the same time, divergence between trait accuracy and state attention. Reassuringly, state auditory attention was not predicted by any of the trait variables (trait interoceptive accuracy: *b* = 0.10, s.e. = 0.20, *t*_100_ = 0.50, *p* = 0.622, 95% CI [−0.29, 0.49]; trait anxiety: *b* = 0.003, s.e. = 0.01, *t*_99_ = 0.27, *p* = 0.786, 95% CI [−0.02, 0.03]; trait depression: *b* = −0.02, s.e. = 0.02, *t*_101_ = −0.86, *p* = 0.390, 95% CI [−0.06, 0.03]). In particular, trait interoceptive attention did not predict state auditory attention (*b* = 0.19, s.e. = 0.17, *t*_101_ = 1.12, *p* = 0.265, 95% CI [−0.14, 0.52]), suggesting that the trait interoceptive attention measures were capturing more than individual differences in attention more generally.

### (e) Relationships between trait interoception and state emotional experience

As a supplementary analysis, we examined the extent to which trait variables predicted state emotional experience. We used a series of multi-level regression models in which all the trait variables (interoceptive attention, interoceptive accuracy, depression and anxiety) were entered as simultaneous level-2 independent variables predicting level-1 emotional experience: (1) negative valence, (2) tense arousal, and (3) fatigue.

Negative valence was significantly positively predicted by trait interoceptive attention (*b* = 0.35, s.e. = 0.16, *t*_99_ = 2.22, *p* = 0.029, 95% CI [0.04, 0.66]) and trait anxiety (*b* = 0.03, s.e. = 0.01, *t*_98_ = 2.76, *p* = 0.007, 95% CI [0.008, 0.52]) but not by trait interoceptive accuracy or trait depression. Similarly, tense arousal was significantly positively predicted by trait interoceptive attention (*b* = 0.37, s.e. = 0.15, *t*_103_ = 2.47, *p* = 0.015, 95% CI [0.07, 0.66]) and trait anxiety (*b* = 0.03, s.e. = 0.01, *t*_102_ = 2.46, *p* = 0.015, 95% CI [0.005, 0.05]) but not by trait interoceptive accuracy or trait depression. Fatigue was significantly positively predicted by trait anxiety only (*b* = 0.02, s.e. = 0.009, *t*_100_ = 2.44, *p* = 0.016, 95% CI [0.004, 0.05]).

## 4. Discussion

This experience-sampling study aimed to examine (1) variation in state self-reported interoceptive attention across 21 different signals; (2) the relationship between state interoceptive attention and concurrent emotional experience, and (3) the relationship between state and trait aspects of interoceptive attention and accuracy. In line with predictions and previous literature, a substantial proportion of variance in state interoceptive attention (44%) was attributable to within-person variability [57]. Across individuals, state interoceptive attention was engaged only 20% of the time, far less frequently than exteroceptive auditory attention (83% of the time). Extending previous literature, we observed that within-person variability differed across interoceptive signals, with sexual arousal, pain, itch, muscles and temperature showing the lowest within-person variability and wind, blood sugar, sneeze and hunger showing the greatest. In line with our second prediction, both increased negative valence and fatigue (but not tension) were associated with greater state interoceptive attention over time. Importantly, this was specific to interoceptive attention, with attention to exteroceptive (auditory) signals showing a distinct pattern in our models (i.e. heightened auditory attention was linked with lower levels of negative valence, tension and fatigue). Finally, we provide the first multiple-time-point evidence for models of interoception in which state and trait measures of interoceptive attention are associated with each other but not with self-reported trait interoceptive accuracy [64]. The implications of these results are discussed below.

The observation of substantial within-person variability in interoceptive attention is consistent with previous literature indicating state effects on several aspects of interoception (both attention and objective accuracy; [57,75]). Given that previous research typically assesses both interoceptive attention and accuracy on a single occasion, our findings of within-person variability question the conceptualization and operationalization of interoception as solely a trait and suggest that to accurately capture individual differences in interoceptive ability, future research should also measure aspects of variability and employ multiple-time-point assessments [24,33].

Notably, we provide the first estimate of rates of interoceptive attention (approx. 20% in everyday life), which were far lower than rates of exteroceptive attention to auditory signals (approx. 83%). This rate of interoceptive attention is also far lower than rates of other types of internal attention (e.g. studies of mind-wandering typically report rates of 30–60% in daily life; [48,76–80]. We observed that, when asked how much attention was focused on interoceptive signals, participants rarely said ‘all of the time’. This may reflect the adaptive nature of interoceptive attention, whereby deviations from homeostatic ‘set-points’ are quickly brought to conscious attention to prompt action to return to those set-points (e.g. drinking water when thirsty), with information from bodily signals otherwise unattended.

Overall low rates of interoceptive attention, and within-person variability, may thus be considered a positive, adaptive, characteristic of the system. However, what accounts for variability across interoceptive signals, between individuals and within individuals, remains unclear. Although participants were directed not to consider whether signals were objectively occurring in the body, it is likely that variability may reflect the length of time that signals are present (e.g. objectively occurring in the body), the extent to which this occurrence represents a deviation from homeostatic set-points, as well as the amount of attention paid to them. For example, certain signals—like an urge to cough—could be relatively short-lived or persisting, with the objective occurrence of signals likely varying both within (e.g. owing to short-term illness) and across individuals (e.g. owing to chronic illness), with greater and lesser variation from homeostatic set-points in the former and latter, respectively. Future research may therefore consider assessing the length of time that different signals objectively occur, as well as how typical the presence of the signal is for the individual, to examine the extent to which these factors contribute to variability across signals, between and within individuals. However, variability may also reflect attentional allocation not coupled to the objective occurrence of signals. What underlies such between- and within-person variability (both heightened and reduced attentional allocation) remains a question for future research. It is conceivable that certain conditions (e.g. chronic pain, eating disorders, panic) are associated with both increased and decreased attentional allocation to specific bodily signals [17]. While we did not observe an association between trait anxiety and attention to internal signals (both state and trait), as might have been expected given previous literature [38], it is notable that our measure of trait anxiety—the STAI—contains many items that may capture depression (see [81]). Future research should seek to examine the source(s) of variability within and across individuals, overall and across specific interoceptive signals, considering the assessment period (e.g. more granular timings than ver the last 5 min), the presence of the signals, their duration and their typicality, as well as the contribution of state and trait factors that may account for variability.

In line with theories of emotion suggesting an important role for interoceptive perception in the generation of emotional experience, we observed concurrent associations between state interoceptive attention and two measures of core affect (negative valence and energetic arousal/fatigue) in everyday life. These findings are consistent with previous literature using single-time-point laboratory assessments of interoceptive accuracy (e.g. [29]) and evidence linking emotional experience to physiological signals [9–13]. Indeed, taken together with previous evidence, our data strengthen the empirical evidence base to support the theory that interoception and emotional experience are linked mechanistically. Our results show that emotional experience may not be driven only by the mere presence of interoceptive signals or one's ability to perceive them accurately, but also, crucially, by the extent of *attention* captured by or allocated to internal signals. Importantly, although ‘better’ interoception (typically higher accuracy) is often considered optimal—forming the basis of contemporary protocols to improve interoceptive ability with training (e.g. [82])—the observation that increased interoceptive attention was associated with negative valence and fatigue (but not tension) suggests that both too little (e.g. where important information is missed) *and* too much attention to internal signals may be maladaptive [33,83] and underscores the need to dissociate metrics of interoceptive accuracy and attention [38,41].

Crucially the associations with emotional experience were specific to bodily signals—with auditory attention associated with more positive valence and less fatigue and tension, again supporting the notion that preoccupation with interoceptive signals, compared with exteroceptive signals, may be maladaptive. Indeed, attention to exteroceptive (auditory) signals was associated with more positive emotional experience, which may in part be driven by daily activities (e.g. conversation, listening to music) thought to promote wellbeing (e.g. [55,84–86]). As with interoceptive attention, future research examining the time course of associations (as well as considering attention to specific exteroceptive auditory signals—e.g. music versus ambient noise) may be beneficial for better understanding these relationships.

Considering the relationship between state and trait aspects when states were averaged over time points, trait levels of anxiety and depression were significantly positively correlated with all the state mood variables as expected (see electronic supplementary material). When examined together, trait interoceptive attention (but not trait interoceptive accuracy) was associated with state emotional experience (specifically tension and negative valence), with trait anxiety (but not depression) also associated with all state emotional experience variables. While the associations between trait interoceptive attention (but not accuracy) and emotional experience may suggest that attention to internal signals is more important for emotional experience than for accuracy, we should note that the accuracy questionnaire provides only an assessment of beliefs about interoceptive accuracy, rather than objective performance. While a useful variable for examining specificity (because bodily signals are matched across the IAS and IATS; [41]), beliefs about interoceptive accuracy do not always predict objective behavioural performance (e.g. [24,38,87]). Taken together with previous research linking laboratory-based measures of interoceptive accuracy to emotional experience in daily life (e.g. [29]), as well as evidence linking beliefs regarding accuracy to emotional awareness (e.g. alexithymia; [38]), our findings suggest that interoceptive attention (both state and trait self-reported) and the objective accuracy of interoceptive perception may be important for emotional *experience*, while beliefs regarding accuracy may be more relevant for emotional *awareness*.

Finally, examination of the correspondence between trait and state measures of interoceptive attention and accuracy extends our understanding of the structure of interoceptive abilities. Consistent with previous research and theoretical models of interoception, self-reported trait measures of interoceptive accuracy and attention were not correlated [38,41]. Importantly, the state measure of interoceptive attention showed a small but significant association with trait interoceptive attention, but not accuracy, a pattern of results consistent with existing models [64]. Crucially these findings were specific to state interoceptive attention, with no significant association between trait measures of interoception and state exteroceptive (auditory) attention. Overall, these data support the importance of distinguishing between interoceptive accuracy and attention in future research. Although trait-to-state associations were small—perhaps unsurprisingly given evidence of within-person variability in interoceptive attention and potential bias in retrospective self-report [65]—this finding further highlights the utility of examining aspects of interoception across multiple occasions.

Some limitations of this study must be acknowledged. First, these data cannot be used to determine causality. While theories of emotion typically predict a directional association between the presence of bodily signals and emotions (e.g. [1–6]), bi-directional and cyclical relationships are plausible and included in some models [88]. Indeed, it is possible that certain emotional states cause attention to be directed towards certain bodily states [88]. Second, demand characteristics (i.e. participants changing their responses based on what they think the research is about) may play some role in observed associations. Since participants rated their attention to interoceptive/ exteroceptive signals first and emotional experiences second, this protocol may have led participants to pay increased attention to intero/exteroceptive signals and/or use these attention states to make inferences about felt emotional experience. This may be especially relevant for some interoceptive signals that were intrinsically negative (e.g. pain) or positive (e.g. pleasant touch), which may have biased valence ratings (see electronic supplementary material, §S2.1 for how attention to the different interoceptive signals was related to emotional experience). Future research may benefit from varying the order in which questions are presented, using less affectively laden interoceptive attention questions, and exploiting wearable technologies to trigger experience-sampling questions based on deviations in physiological states (e.g. heart rate; [89]). Such work would also enable uncoupling of endogenous versus exogenous (signal present) attentional processes and permit exploration of potential bi-directional associations between emotion and interoceptive attention. Finally, we should note that these data can only be used to make inferences regarding explicit (conscious) attention to internal signals. Some contribution of internal signals to emotion may also occur at a level not accessible by self-report, meaning that our results may underestimate the contribution of interoception to emotional experience.

In summary, our results support previous models of interoception regarding the relationship between interoceptive aspects [64] and for the first time highlight associations between state emotional experience and interoceptive attention over time. State interoceptive attention varied both within and across individuals, with the contribution of within-person variability differing across interoceptive signals. Future research should examine the source(s) of within- and between-person variability in interoceptive and exteroceptive attention and its relationship with emotional experience. Such research may benefit from using stimulus-locked ESM, protocols assessing the presence of physiological signals in combination with behavioural assessments of interoceptive accuracy. Doing so will be useful for examining the contribution of various aspects of interoception to emotional experience and understanding how different aspects of interoception are related to each other.

## Data Availability

Data are available at https://osf.io/49wbv/?view_only=2188768b1b92472698671f6a6dfc859f [90]. Supplementary material is available online [91].

## Endnotes

1 A much earlier experience-sampling study [63] that should be noted examined whole-body awareness during the past 10 min and the degree to which awareness was negative or positive. The study did not measure emotional experience *per se* but did demonstrate that attention to specific body parts or functions was perceived more negatively, and also that women (compared with men) tended to have negative perceptions of bodily awareness in daily life.

2 The first 38 participants completed 7 days of ESM and the remaining 69 completed 5 days. Study duration was reduced owing to participant feedback regarding burden. Interoceptive attention across the study did not meaningfully differ between groups (*b* = 0.02, s.e. = 0.04, *t*_103_ = 0.44, *p* = 0.662, 95% CI [−0.07,0.11]).

3 Scales with Cronbach's alpha between 0.70 and 0.95 are considered to have good internal consistency [68].

4 Note that results and conclusions were unchanged when predictors were entered independently in the models.
